# ‘Distribution of Hepatitis C Virus genotypes in northern Greece in the last decade: descriptive analysis and clinical correlations’

**DOI:** 10.1017/gheg.2019.4

**Published:** 2019-09-02

**Authors:** G. Gioula, E. Sinakos, E. Gigi, I. Goulis, T. Vasiliadis, F. Minti, E. Akriviadis

**Affiliations:** 1 2nd Department of Internal Medicine, Laboratory of Hepatology, Hippokratio Hospital, Aristotle University of Thessaloniki, Thessaloniki, Greece; 2 4th Department of Internal Medicine, Hippokratio Hospital, Aristotle University of Thessaloniki, Thessaloniki, Greece; 3 2nd Department of Internal Medicine, Hippokratio Hospital, Aristotle University of Thessaloniki, Thessaloniki, Greece; 4 3rd Department of Internal Medicine, Papageorgiou Hospital, Aristotle University of Thessaloniki, Thessaloniki, Greece

**Keywords:** Genotypes, Greece, hepatitis C virus

## Abstract

Hepatitis C virus (HCV) represents a major public health problem, while the identification of a HCV genotype is clinically very important for therapy prescription. The aim of the present study was to determine the HCV genotype distribution patients from northern Greece with HCV RNA positive viral load and to identify whether there is a shift in this distribution, during 2009–2017. The study was performed on 915 HCV positive patients and according to the results, genotype 3 was the most prevalent genotype (*Ν* = 395, 43.2%) followed by genotype 1 (*Ν* = 361, 39.5%). Regarding the gender of the patients, genotype 1 was mostly detected in women. Moreover, genotype 1 was associated with higher viral loads, while genotype 3 was most frequently detected in patients with a history of intravenous drug use. In conclusion, our results show that genotype 3 is the most prevalent genotype in Greece during the last decade as opposed to older epidemiological studies, likely due to intravenous drug use becoming the major source of infection.

## Introduction

Hepatitis C virus (HCV) represents a major public health problem. It is a principal cause of chronic liver diseases including liver fibrosis, liver cirrhosis and hepatocellular carcinoma, as after acute infection, around 50–80% of HCV patients will develop chronic infection [1]. According to the WHO, an estimated 71 million people have chronic hepatitis C and over 399 000 patients yearly die due to HCV-related disorders [2]. In May 2016, The World Health Assembly adopted the first ‘Global Health Sector Strategy on Viral Hepatitis, 2016–2021’. This strategy has the vision of eliminating viral hepatitis as a public health problem and this is encapsulated in the global targets of reducing new viral hepatitis infections by 90% and reducing deaths due to viral hepatitis by 65% by 2030 (http://www.who.int/mediacentre/factsheets/fs164/en/).

HCV is an enveloped virus having positive single-stranded RNA that was first discovered in 1989, belonging to the virus family Flaviviridae. The virus exhibits an extraordinarily high degree of genetic diversity and its strains are classified into seven recognised genotypes on the basis of phylogenetic and sequence analyses of whole viral genomes. HCV strains, belonging to different genotypes, differ at 30–35% of nucleotide sites, while within each genotype, HCV is further classified into 67 confirmed and 20 provisional subtypes [3]. Available data indicate that genotypes 1 and 3 have a worldwide distribution and account for the majority of HCV infections in Europe. Genotype 1 is more frequent in older patients, while genotype 3 is more common between intravenous drug users [4]. The estimation of current prevalence of Hepatitis C in Greece ranges from 0.5% to 2% [5], while an epidemiological study, which cites data from a phone survey, reports an age-adjusted anti-HCV prevalence of 1.79% (1.87% high-risk individuals-adjusted) for the population of the study (ages 18–70), and a total estimated prevalence of 1.47% [6].

Identification of the HCV genotype clinically is very important, before prescribing therapy. Although pan-genotypic regimens, among other equally effective treatment options, are currently recommended, HCV genotyping still remains relevant [7]. Apart from different national policies for access to treatment with direct acting antivirals, the knowledge of each patient's genotype before treatment is essential as differences in the treatment effectiveness (especially for genotype 3) and the genotype prevalence according to various sub-populations exist at the present time. Therefore, it is clear that a better knowledge of the epidemiology of HCV and of distribution of its genotypes could substantially contribute to an effective control of this disease, especially by focusing screening strategies on patients at risk of disease progression, in order to get them into treatment earlier. Additionally, in the past few years there has been a global increase in genotype 3 and a decrease in genotype 1 prevalence [8, 9]. According to recent data, this is mainly correlated with changes in the mode of HCV transmission [9, 10]. Blood safety and improvement in infection control practices have contributed to the fact that people who inject drugs (PWID) become not only the main risk factor for HCV transmission but also to change the genotype distribution among patients with HCV hepatitis [8–10].

The aim of the present study was to determine the HCV genotype distribution of HCV RNA positive viral load in patients from northern Greece and to identify whether there is a shift in this distribution, between 2009 and 2017.

## Materials and methods

The study, which was a retrospective analysis, was performed on 915 HCV positive patients, whose serum samples were collected from January 2009 to December 2017. No data were available during 2015–2016, due to unavailability of the appropriate reagents in the laboratory, because of the financial crisis. Moreover, there is a marked reduction in the number of HCV positive tests in 2014 and 2017 (making up only 5% of the total sample), which was also due to unavailability of the appropriate reagents in the laboratory.

Complete medical history was evaluated and the following demographic and disease characteristics were recorded: age, gender, ethnicity, and possible source of infection. The study was conducted in the Laboratory of Hepatology of 2nd Department of Internal Medicine of Aristotle University of Thessaloniki, in collaboration with the Hepatology Departments of 2nd Department of Internal Medicine, 4th Department of Internal Medicine of Aristotle University of Thessaloniki and 3rd Department of Internal Medicine of Aristotle University of Thessaloniki.

The diagnosis of HCV was confirmed by the detection of HCV RNA in the patients' serum by the Versant HCV RNA Assay (kPCR) and genotyped by the Versant HCV Genotype 2.0 assay (Lipa), both by Siemens, according to the manufacturer's instructions.

### Statistical analysis

Data analysis was conducted with IBM SPSS Statistics Version 23. In order to detect significant associations in the data (genotypes, gender and ethnicity), Pearson's χ^2^ test for independence and Fisher's exact test with confidence interval (CI) 95% were implemented. Results with a *p* value of 0.05 were significantly considered. Additionally, adjusted residuals were calculated to further strengthen the associated data.

## Results

### Genotype distribution

**Fig. 1.**
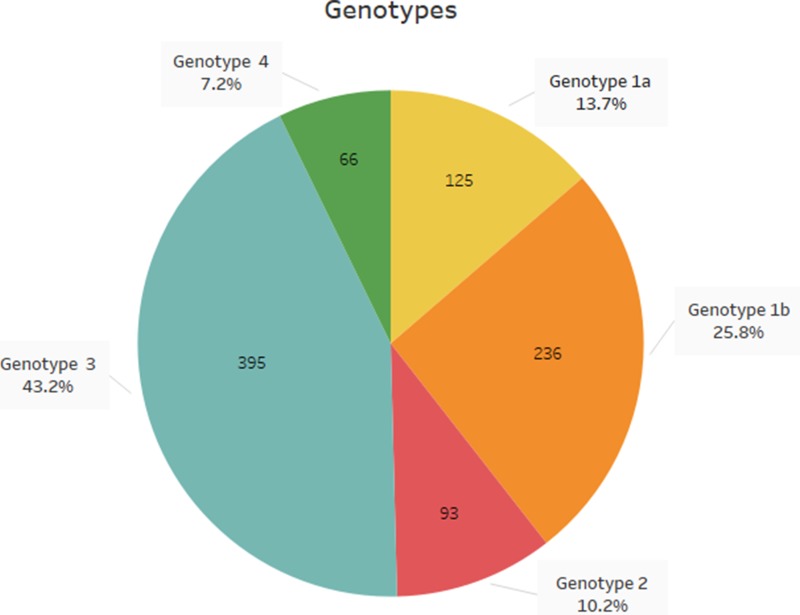
Distribution of HCV genotypes in northern Greece, during the last decade.

The study population included 705 men and 210 women (median age 48.5 years), while 829 of the patients were Greek and the remaining 86 were immigrants, mostly from the former Soviet Union countries. According to the results, genotype 3 was the most prevalent genotype (*Ν* = 395, 43.2%) followed by genotype 1 (*Ν* = 361, 39.5%) (genotype 1a 13.7% and genotype 1b 25.8%). Genotype 2 was detected in 10.2% (*Ν* = 93) of the patients, while genotype 4 in 7.2% (*Ν* = 66) of them; genotypes 5 and 6 were not detected among the study population (Fig. 1). The distribution of HCV genotypes during the years 2009–2017 is shown in Table 1. During 2015–2016 there are no data available due to unavailability of the appropriate reagents in the laboratory.

**Table 1.** Distribution of HCV genotypes in Northern Greece during the last decadr.

Ethnicity			Genotype detail					Total
			1a	1b	2	3	4	
Foreign			25	24	9	25	3	86
Greek	Gender	Female	7	73	16	61	16	173
		Male	93	139	68	309	47	656
	Greek total		100	212	84	370	63	829
Total			125	236	93	395	66	915

### Clinical associations

Regarding the gender of the patients, genotype 1 was mostly detected in women (47.6% *v*. 32.9%), while genotype 3 was most frequently present in men (46.2% *v*. 37.0%) [*p* = 0.005, 95% CI (0.004–0.007)]. The frequency of genotype 2 was similar in both genders (men/women: 10.1/10.5%), while genotype 4 was slightly more prevalent in women (9.0% *v*. 6.7%), but this was not statistically significant. Regarding the ethnicity of the study population, genotype 1 was detected in a higher proportion in immigrants (57%), while in Greek patients, genotype 3 was most frequently detected (44.6%) [*p* = 0.004, 95% CI (0.003–0.006)]. The frequency of genotype 2 was similar between patients with different ethnicities (Greek patients 10.1% *v*. immigrants 10.5%) and genotype 4 was detected in lower proportion in immigrants (3.5%) than Greeks (7.6%) in this study population. Moreover, patients infected by genotype 1 (median, 50 years) were older than patients infected by genotype 3 (median, 33.5 years), but without any statistical significant difference. Although genotype 1 was associated with higher viral loads and genotype 3 was most frequently detected in patients with a history of intravenous drug use, this was not a finding with a statistical significant difference. Concerning genotypes 1a and 1b, 1a was mostly detected in men (86.4%), while genotype 1b was also most prevalent in men population, but in a lower degree (64.8%), without also any statistical difference.

## Discussion

The study and understanding of the HCV genotype distribution is crucial as a part of a molecular clue for the spread of HCV. Moreover, it is well-documented that genotype distribution is associated with the mode of transmission. Over the past few decades, there have been remarkable changes in hepatitis C epidemiology due to changes in the predominant route of transmission [11]. More specifically, over the past few years, there has been observed a global increase in genotype 3 and a decrease in genotype 1. According to data, this is mainly correlated with changes in the mode of HCV transmission, since PWID has become the main risk factor for HCV transmission [10, 12, 13]. However, challenges in HCV prevention remain as PWID still face several barriers in the treatment cascade despite the improvements that have been made worldwide.

The main finding of the present study is the substantial change in genotype distribution of HCV infection over the last years. The results revealed that HCV genotypes 1 and 3 accounted for the vast majority of HCV infections in patients from northern Greece, while genotype 3 was the most common one, followed by genotype 1.

The results of a similar multicentre national Greek study that was conducted in 2011, demonstrated that the distribution for genotypes 1, 2, 3 and 4 was 45.1, 7, 34 and 13.9%, respectively, while genotype 1 was more common in older people, women and patients with transfusion-related hepatitis [14]. In the same study a trend in increase of genotype 3 infection in intravenous drug use during the recent years was also observed.

Moreover, recent retrospective analysis conducted in Greece, from six liver clinics, during the period from 1 May 2014 to 31 May 2017, in PWID suggested that HCV genotype 3 remains the predominant genotype among PWID (61%) [15].

Another finding of the present study was the older age of patients infected by genotype 1 than this of patients infected by genotype 3, which is probably due to the fact that younger HCV positive patients belong to the group of PWID acquired infection, while the older may have been infected by HCV through blood transfusion or other sources. According to the results of a multicentre national Greek study, a significant difference of genotype distribution based on age at infection was observed. Genotype 1 was less common in patients <25 years of age, than in those ⩾25 years old (33.7% *v* 48.2%), whereas, genotype 3 was less common in patients ⩾25 years old (31.0% *v*. 49.0%) [14]. It is worth mentioning that the risk of transfusion-related HCV-hepatitis progressively declined during 1980s and 1990s possibly due to the implementation of an all-volunteer blood donor system and the effective virus-inactivation procedures for blood derivatives. It should be noted that in Greece the blood system started screening donations with the first generation EIA in 1992, whereas the 2nd and 3rd generation EIA were introduced in 1994 and 1999, respectively [14].

Similar studies in the Balkan area showed the following results: in Slovenia subtype 1b was the most prevalent among chronic hepatitis C patients infected by unknown cause [16]. In Serbia and Montenegro, the genotypes 1b and 3a predominate in patients with chronic HCV infection, while in the Former Yugoslavian Republic of Macedonia, genotype 1 was predominant in the group of haemodialysis patients, while genotype 3 was predominant in intravenous (IV) drug users [17, 18]. Moreover, in a study conducted in St Petersburgh, among 387 intravenous drug users (IDUs), genotype 3 predominated (56.9%), followed by genotype 1 (41.1%) [19].

In the present study, HCV genotype distribution differed significantly by gender. The same observation was already made in previous studies conducted in Greece, with genotype 1 being more frequent in women (55.9% *v*. 37.8%), and genotype 3 more frequent in men (43.0% *v*. 20.6%) [6, 14].

Regarding the ethnicity, the same difference in the genotype distribution between Greeks and immigrants has been reported in a previous study (Greek/immigrants: 45.2% *v*. 49.8% and 34.6% *v*. 22.6% for genotypes 1, and 3 respectively; *p* = 0.001) [14]. Moreover, taking into account that the majority of immigrants came from Soviet Union countries and mostly Georgia, we observe from previous studies in the specific country that genotype 1b was the predominant one (59%) [20].

There are, however, some limitations in the present study: (a) Possible route of transmission was unknown for a substantial number (~20%) of the study population. (b) No data were available during 2015–2016, due to unavailability of the appropriate reagents in the laboratory, because of the financial crisis. (c) There is a marked reduction in the number of HCV positive tests in 2014 and 2017 (making up only 5% of the total sample). This was also due to unavailability of the appropriate reagents in the laboratory and as a result, clinicians did not send serum samples for genotyping in the laboratory, due to delay in the test results. This may suggest a sample bias. (d) As is a retrospective cohort study, there is not an equal distribution of the factors, as the exposure and the outcome variables are collected before the study has been initiated. Thus, the measurements may not be very accurate or according to our requirements. (c) ‘In addition, the timing of HCV infection in foreign patients with regard to their arrival in Greece is not clear making the comparison between Greek and foreign patients to some extent ambiguous'.

In conclusion, our results show that the epidemiology of HCV infection is changing in Greece. An increase of genotype 3 has been observed with a simultaneous reduction of genotype 1, probably because of the changes in the mode of transmission during the recent decades. Furthermore, due to the rising prevalence of HCV among IDUs, this population should become the focus of interventions for the prevention of HCV infection and a more systematic treatment of hepatitis C in the drug users is needed, independent of concerns about adherence to treatment and the postulated high risk of re-infection.
